# Heat-Induced Oxidation of the Nuclei and Cytosol

**DOI:** 10.3389/fpls.2020.617779

**Published:** 2021-01-12

**Authors:** Richa Babbar, Barbara Karpinska, Anil Grover, Christine H. Foyer

**Affiliations:** ^1^School of Biosciences, College of Life and Environmental Sciences, University of Birmingham, Birmingham, United Kingdom; ^2^Department of Plant Molecular Biology, University of Delhi South Campus, New Delhi, India

**Keywords:** epigenetics, heat shock proteins, reactive oxygen species, redox-sensitive green fluorescent protein, oxidation

## Abstract

The concept that heat stress (HS) causes a large accumulation of reactive oxygen species (ROS) is widely accepted. However, the intracellular compartmentation of ROS accumulation has been poorly characterized. We therefore used redox-sensitive green fluorescent protein (roGFP2) to provide compartment-specific information on heat-induced redox changes of the nuclei and cytosol of Arabidopsis leaf epidermal and stomatal guard cells. We show that HS causes a large increase in the degree of oxidation of both compartments, causing large shifts in the glutathione redox potentials of the cells. Heat-induced increases in the levels of the marker transcripts, heat shock protein (*HSP*)*101*, and ascorbate peroxidase (*APX*)*2* were maximal after 15 min of the onset of the heat treatment. RNAseq analysis of the transcript profiles of the control and heat-treated seedlings revealed large changes in transcripts encoding HSPs, mitochondrial proteins, transcription factors, and other nuclear localized components. We conclude that HS causes extensive oxidation of the nucleus as well as the cytosol. We propose that the heat-induced changes in the nuclear redox state are central to both genetic and epigenetic control of plant responses to HS.

## Introduction

Heat stress (HS) is a major threat to current and future agriculture. Future increases in atmospheric CO_2_ levels and associated global warming are predicted to amplify the impact of HS on crops such as wheat, rice, and maize that form the basis for human nutrition. Climate change models predict that mean ambient temperatures will increase between 1.8 and 5.8°C by the end of this century as well as the frequency of heat waves. Ensuing the sustainability yield of all major crops in the global warming era is a major challenge faced by plant scientists (Easterling and Apps, 2005). Plant responses to HS are very well characterized. HS has drastic impact on seed germination, radicle emergence, plumule growth, and seedling growth (Toh et al., 2008; Piramila et al., 2012). Reproductive organs, especially the male reproductive tissues (Giorno et al., 2013) are highly sensitive to HS-induced loss of function, leading to a significant reduction in yield (Warland et al., 2006; Ahamed et al., 2010). Exposure to HS causes flower abortion, spikelet sterility, and decreased pollen germination (Sato et al., 2006). Photosynthesis is also sensitive to heat-induced inhibition (Berry and Bjorkman, 1980; Wise et al., 2004; Rossi et al., 2017; Wang et al., 2018). In particular, the activation of ribulose-1, 5-bisphosphate carboxylase oxygenase (Rubisco) by Rubisco activase is heat sensitive (Portis, 2003; Sage et al., 2008; Perdomo et al., 2017), as are starch and sucrose synthesis (Sumesh et al., 2008). Thylakoid membrane stabilization is also an important response to HS (Zhang and Sharkey, 2009; Niinemets, 2018), as is the induction of molecular chaperones, the accumulation of solutes, and changes in gene expression through mitogen-activated kinase and calcium-dependent protein kinase cascades (Wahid et al., 2007; Dietz et al., 2016).

The accumulation of reactive oxygen species (ROS) coupled to the induction of heat shock proteins (HSPs) are characteristic features of the heat shock response (Suzuki and Katano, 2018). The accumulation of ROS is a key trigger for plant stress responses that can lead either to acclimation or programmed cell death depending on the signaling pathways involved (Baxter et al., 2014; Noctor et al., 2018). The HS-induced accumulation of H_2_O_2_ is a key trigger in regulation of the expression of HSPs. The expression of HSPs is governed by heat shock factors (Hsfs), which are transcription factors that bind to the heat shock promoter elements (HSEs) in the *HSP* genes (Liu and Charng, 2013). Arabidopsis has 21 Hsfs of which the heat-inducible class A Hsfs, *HsfA2*, *HsfA7a*, and *HsfA3*, and class B Hsfs, *HsfB1*, *HsfB2a*, and *HsfB2b*, play an essential role in acquired thermotolerance (Meiri and Breiman, 2009). ROS accumulation induces the expression of Hsfs and HSPs accumulation (Zhang et al., 2009; Banti et al., 2010). Hsfs such as HsfA1b and HsfA2 are involved in the regulated expression of the marker gene, *ascorbate peroxide 2* (*APX2*), which is a marker for the HS response (Schramm et al., 2006; Ogawa et al., 2007). Thermotolerance is associated with changes in phytohormones such as salicylic acid (SA) that amplify H_2_O_2_ production and the expression of *HSP* genes.

In natural environments, the exposure to HS often occurs in combination with other stresses such as high light and drought (Choudhury et al., 2017). However, the combination of HS and high light leads to an increase in jasmonic acid (JA) levels and the subsequent activation of transcriptional responses that are distinct from the responses to the single stress treatments (Balfagón et al., 2019). Moreover, *APX2* expression was only shown to be regulated by high light if there was a simultaneous increase in temperature (Huang et al., 2019).

Although there is overwhelming evidence in support of HS-induced ROS accumulation, little information is available concerning the status of these compounds in the cellular compartments where ROS are produced. The ROS-induced oxidation of the cellular antioxidant reduced glutathione (GSH) to glutathione disulfide (GSSG) causes a shift of the glutathione redox potential (EGSH) toward less negative values, a process that is considered to be hallmark of exposure to stress (Noctor et al., 2011). The application of *in vivo* imaging techniques such as redox-sensitive green fluorescent proteins (roGFPs) provides compartment-specific information on the degree of oxidation and the glutathione redox potential (Meyer et al., 2007; Schwarzländer et al., 2008; García-Quirós et al., 2020). Genetically-encoded roGFP2-based sensors are robust and highly reliable probes for monitoring cellular oxidation. The fusion of human glutaredoxin-1 to roGFP2 (Grx1-roGFP2) increases the speed of roGFP2 oxidation by GSSG (for EGSH), while fusing the yeast glutathione peroxidase, Orp1, to roGFP2 enhances the specificity to H_2_O_2_. The H_2_O_2_-specific roGFP2-Orp1 probe has been used to analyze H_2_O_2_ dynamics in the intracellular compartments of Arabidopsis cells (Nietzel et al., 2019; Ugalde et al., 2020). Moreover, studies using Grx1-roGFP2 and roGFP2-Orp1 have demonstrated that exposure to the oxidizing herbicide methyl viologen causes rapid oxidation of the chloroplasts, cytosol, and mitochondria (Ugalde et al., 2020). We have previously used the roGFP2 probe to analyze changes in EGSH in the leaves (Schnaubelt et al., 2015) and flowers (García-Quirós et al., 2020) of mutants that are deficient in glutathione, as well as redox changes occurring in the cell cycle in the primary roots of wild-type (WT) Arabidopsis and mutants that are deficient in the antioxidant, ascorbic acid, and the effects of chloroplast inhibitors on EGSH in the nuclei and cytosol of leaf epidermal cells and stomatal guard cells (Karpinska et al., 2017a). We therefore extended this analysis to explore the effects of HS on the redox states of the cytosol and nuclei of *Arabidopsis thaliana* (Arabidopsis) seedlings in order to provide to further explore the effects of HS on the redox state of different cellular compartments. We provide evidence of extensive heat-induced oxidation of the nuclei as well as the cytosol and discuss how the regulated oxidation of both cellular compartments can influence the cell signaling in both cellular compartments to achieve thermotolerance and survival of HS.

## Materials and Methods

### Plant Material

Seeds of WT *A. thaliana* accession Columbia-0 (Col-0), Col-0 expressing roGFP2 (de Simone et al., 2017), were surface sterilized (70% ethanol, 2% {v/v} bleach, and 0.1% Tween 20) and washed twice with sterile water. The seeds were plated onto half strength Murashige-Skoog (MS) medium containing 1% sucrose and 0.8% agar (pH 5.8). After stratification in a cold room (4°C) for 48 h, the plates were transferred to a growth room maintained at 22 ± 1°C with 16:8 h light and dark regime and a light intensity of 120 μmol m^–2^s^–1^. Agar-grown seedlings were used in the present study because this method has been used in previous studies on HS (Silva-Correia et al., 2014; Tiwari et al., 2020).

### HS Treatments

Heat stress was applied to 5-days-old Arabidopsis seedlings growing on petri plates. The plates were sealed using Petriseal (Diversified Biotech, United States) and exposed to 42°C for 60 min in a water bath. Batches of control seedlings were maintained at 22°C. Each biological replicate consisted of 100 control or heat-stressed seedlings. Three biological replicates were used per timepoint and each experiment was repeated at least three times. Samples were harvested from control and heat-stressed seedlings at the time points shown in the figure legends or immediately after treatment had ended and used in the analysis below.

### Confocal Laser Scanning Microscopy and Image Analysis

For quantitative monitoring of EGSH, we used the roGFP2 variant derived from the original EGFP containing the S65T mutation that has a main excitation peak at 490 nm (Hanson et al., 2004). The roGFP2 form has advantages in less reducing conditions, as occur, for example, in partially glutathione-deficient mutants (Meyer et al., 2007). Control and heat-treated seedlings were placed on a slide in a drop of sterile water. roGFP2 was then interrogated using a Carl Zeiss confocal microscope LSM700 equipped with lasers for excitation at 405 and 488 nm to image oxidized and reduced form of ro-GFP2, respectively. Images were taken with 40x/1.3 Oil DIC M27 lens in a multitrack mode. Ratiometric analyses were performed using ImageJ software^1^. The roGFP2 signal was calibrated at the end of each experiment using standard conditions of incubation with either 2.5 mM dithiothreitol (reduced) or 2 mM H_2_O_2_ (oxidized). Samples for fully reduced and oxidized controls were treated for 10 min with dithiothreitol or H_2_O_2_, respectively. The oxidation degree and glutathione redox potential values were calculated as described by Meyer et al. (2007) and de Simone et al. (2017).

### Nitro Blue Tetrazolium (NBT) and 3,3′-Diaminobenzidine-HCl (DAB) Staining

Staining with nitro blue tetrazolium (NBT) and 3,3′-diaminobenzidine (DAB) was performed essentially as described by Groten et al. (2005). For NBT staining, whole seedlings were vacuum-infiltrated in 10 mM NaN_3_ and stained in 0.5 mg ml^–1^ NBT (Sigma–Aldrich) prepared in 10 mM potassium phosphate buffer (pH 7.8) for 30 min. The reaction was stopped by transferring the seedlings into 90% ethanol at 70°C until all the chlorophyll was removed. The seedlings were photographed under a stereomicroscope (Leica M165-FC; Leica Microsystems, Wetzlar, Germany). DAB (5 mg ml^–1^) in dimethyl sulfoxide (DMSO), diluted 1: 10 with 10 mM sodium phosphate buffer (pH 7.8), was used to detect H_2_O_2_, using a similar protocol.

### RNA Seq

Control and heat-stressed seedlings were harvested in liquid nitrogen and immediately stored at −70°C before further analysis. Total RNA was isolated from 5-days-old Arabidopsis seedlings using RNA purification kit (Sigma–Aldrich, United States) according to the manufacturer’s instructions. RNA quality and quantity was examined by Nanodrop and on the basis of RIN value (RNA integrated number). 1 μg of total RNA was used for the construction of RNAseq libraries. Single-end RNAseq libraries were constructed independently using the RNA Sample Preparation Kit (Illumina, Foster City, CA, United States) according to the manufacturer’s instructions. Sequencing was performed on the Illumina GAII platform. Unpaired 100 bp Illumina reads were aligned using TopHat (v2.0.10) against a Bowtie2 (v2.2.8) index built from the TAIR10 reference sequence to create sequence alignment/map (SAM) files. SAM files were then sorted and converted into binary alignment/map (BAM) alignment files using Samtools (v1.3). The aligned read replicates were counted using feature Counts to create gene-count matrix and tested for differential gene expression using EdgeR in R/Bioconductor. Differentially expressed genes were defined as those showing fold changes of greater than 2 and a false discovery rate (FDR) corrected *p*-value of 0.05 or less (Supplementary Table 1). The datasets presented in this study can be found in online repositories. The names of the repository/repositories and accession number(s) can be found below: NCBI BioProject, accession no: PRJNA669354.

### Real-Time PCR

Real-time qPCR was performed as described previously (de Simone et al., 2017). The abundance of transcripts was measured in samples harvested during the HS treatment at the time points indicated in the figure legends. Total RNA was extracted as described above. RNA reverse transcription and qPCR were performed on an Eppendorf Realplex2 real-time PCR system by one-step RT-PCR using Quantifast SYBR Green RT-PCR Kit (Qiagen), following manufacturer’s instructions. The expression of the genes of interest was initially normalized using four house-keeping transcripts. We have reported the data in terms of one reference gene, actin, which proved to be the most stable in response to the HS treatment. However, the other reference genes gave very similar results. Accessions and primer sequences used are given in Table 1.

**TABLE 1:** Sequences used for qPCR analysis.

Oligo name	Sequence	Gene ID	Annotation
Act2.F	5′-ACCTTGCTGGACGTGACCTTACTGAT-3′	At3g18780	ACTIN 2
Act2.R	5′-GTTGTCTCGTGGATTCCAGCAGCTT-3′		
Apx1.F	5′-GTCCATTCGGAACAATGAGGTTTGAC-3′	At1g07890	ASCORBATE PEROXIDASE 1, cytosolic
Apx1.R	5′-GTTGTCTCGTGGATTCCAGCAGCTT-3′		
Apx2.F	5′-CCCATCCGACCAAACACATCTCTTA-3′	At3g09640	ASCORBATE PEROXIDASE 2, cytosolic
Apx2.R	5′-CCCATCCGACCAAACACATCTCTTA-3		
Apx3.F	5′-CCCAAAATCACATACGCAGACCTGTA-3	At4g35000	ASCORBATE PEROXIDASE 3, microsomal
Apx3.R	5′-AGTTGTCAAACTTCAGCGGCTCTTG-3′		
Apx4.F	5′-CTACTAAATCCGGGGGAGCCAATG-3′	At4g09010	ASCORBATE PEROXIDASE 4, microsomal
Apx4.R	5′-CTCTGTTGCATCACTCCTTCCAAAAT-3′		
Apx-s.F	5′-TGCTAATGCTGGTCTTGTGAATGCTT-3′	At4g08390	ASCORBATE PEROXIDASE, stromal
Apx-s.R	5′-CCACTACGTTCTGGCCTAGATCTTCC-3′		
Apx-t.F	5′-CAGAATGGGACTTGATGACAAGGAAA-3′	At1g77490	ASCORBATE PEROXIDASE, thylakoidal
Apx-t.R	5′-ATGCAGCCACATCTTCAGCATACTTC-3′		
Apx6.F	5′-TGCAAAACGAAATAAGGAAAGTGGTG-3′	At4g32320	ASCORBATE PEROXIDASE 6, cytosolic
Apx6.R	5′-CACTCAGGGTTTCTGGAGGTAGCTTG-3′		
HSP101.F	5′-TTATGACCCGGTGTATGGTG-3′	At1g74310	HEAT SHOCK PROTEIN 101, cytosolic and nuclear
HSP101.R	5′-AGCGCCTGCATCTATGTAAA-3′		

## Results

**FIGURE 1:**
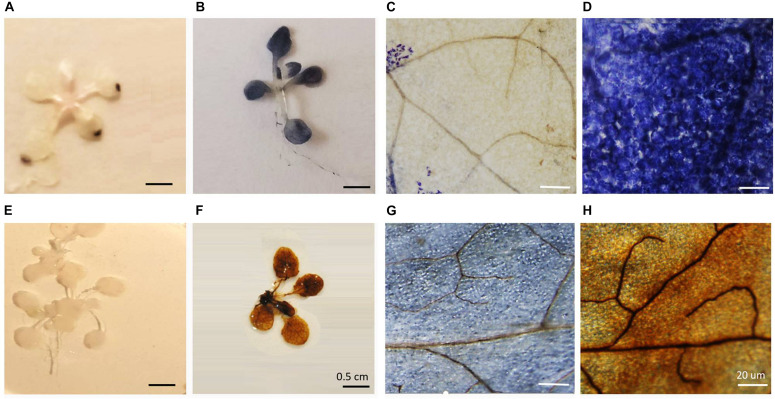
The effects of heat stress on nitrobluetetrazolium **(A–D)** and 3,3′–diaminobenzidine **(E–H)** staining of leaves.

Seven-days-old agar-grown seedlings were subjected to HS by placing the sealed plates in a water bath at 42°C for 1 h (Silva-Correia et al., 2014; Tiwari et al., 2020). Control seedlings were treated in the same way but the water bath was maintained at 22°C. The survival of the HS treatment seedlings was 98%, a value similar to the control and heat-stressed seedlings maintained at 22°C. Following HS, the seedlings were stained with either NBT or DAB. While both the NBT and DAB staining techniques have limitations, as we have discussed previously, these techniques have been widely used to indicate changes in the level of oxidation experienced by stressed organs and tissues (Noctor et al., 2016). In control seedlings (Figure 1A), blue staining was only observed in the tips of the rosette leaves. In contrast, following the HS treatment, the blue staining was observed throughout the rosette leaves (Figure 1B). A higher resolution image of the stained leaves revealed that while relatively few cells in the control leaves had blue staining (Figure 1C), all of the cells contained blue staining after HS (Figure 1D). No DAB staining was observed in the rosettes in the absence of HS (Figure 1E), but the leaves of the heat-stressed leaves showed staining (Figure 1F). Higher resolution images of the stained leaves showed that while some cells in the control leaves had brown staining (Figure 1G) but brown staining was greatly increased around the veins after HS (Figure 1H).

**FIGURE 2:**
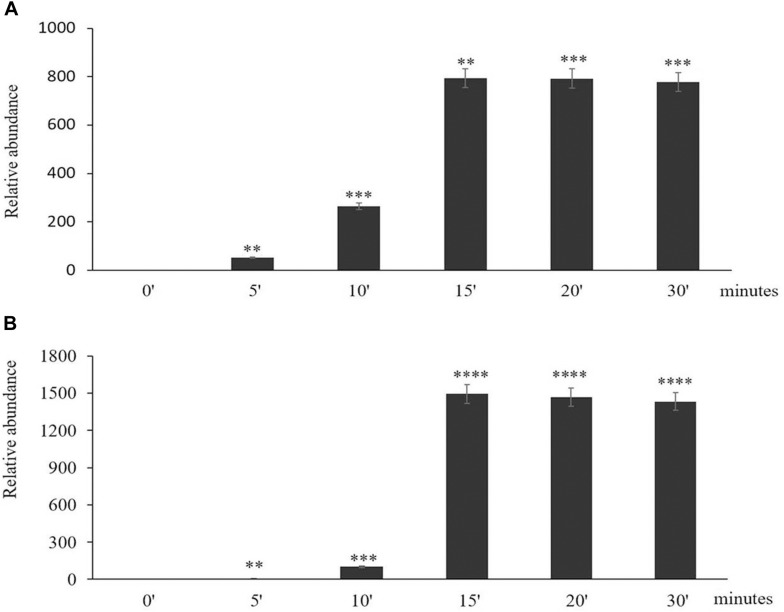
The time course of expression of *Heat Shock Protein* (*HSP*) *101* **(A)** and *Ascorbate Peroxidase* (*APX*)*2* **(B)** in response to heat stress treatment. Asterisks indicate statistical significance (*P* ≤ 0.05) between the treatments. Seedlings were exposed to heat stress for 0 (control), 5, 10, 15, 20, and 30 min. Values are means ± SD (*n* = 3 independent biological replicates each consisting of 100 seedlings). Asterisks indicate significant differences between control and heat-stressed plants according to the Student’s *t*-test (^∗^*p* < 0.05; ^∗∗^*p* < 0.01; ^∗∗∗^*p* < 0.001; and ^****^p < 0.0001).

**FIGURE 3:**
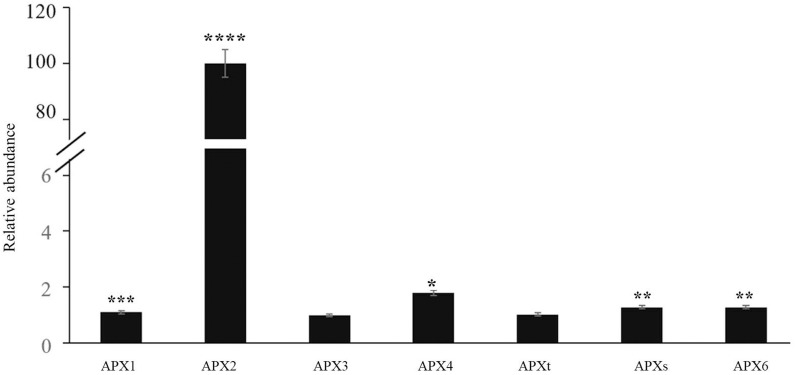
The abundance of transcripts encoding all the Arabidopsis ascorbate peroxidase (APX) family members in control and heat-stressed seedlings. The APX gene family included three *cytosolic* (*APX1*, *APX2*, *APX6*) forms, two chloroplast forms (a stromal form *APX*_*s*_ and a thylakoid form *APX*_*t*_), and two microsomal (*APX3*, *APX4*) forms. Values are means ± SE (*n* = 3 independent biological replicates each consisting of 100 seedlings). Asterisks indicate significant differences between control and heat-stressed plants according to the Student’s *t*-test (*p < 0.05; **p < 0.01; ***p < 0.001; and ****p < 0.0001).

The time course of expression of the marker genes *HSP101* (Figure 2A) and *APX2* (Figure 2B) showed that the levels of transcripts increased rapidly after the onset of the HS treatment and was maximal after 15 min, remaining at a high level thereafter. Moreover, an analysis of the abundance of transcripts of seven members of the Arabidopsis APX family revealed that only the levels of the *APX2* transcripts were increased as a result of the HS treatments and the levels of other APX transcripts were nearly similar in control and heat-stressed seedlings (Figure 3).

**FIGURE 4:**
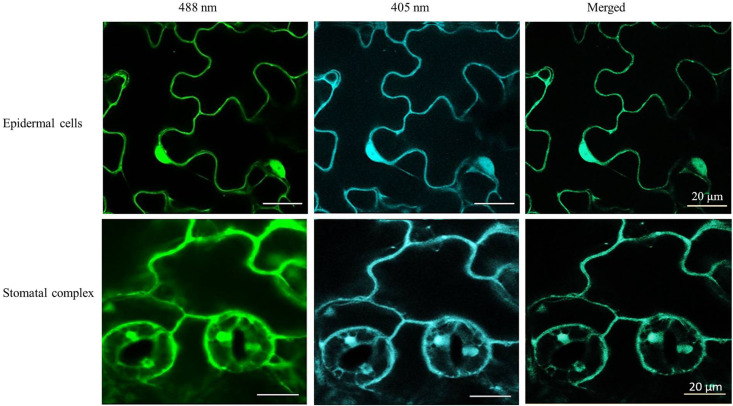
Examples of typical ro-GFP2 fluorescence images of the epidermal cells and stomatal guard cells on leaves exposed to heat stress (42°C) for 1 h. Scale bar is 20 μm.

**FIGURE 5:**
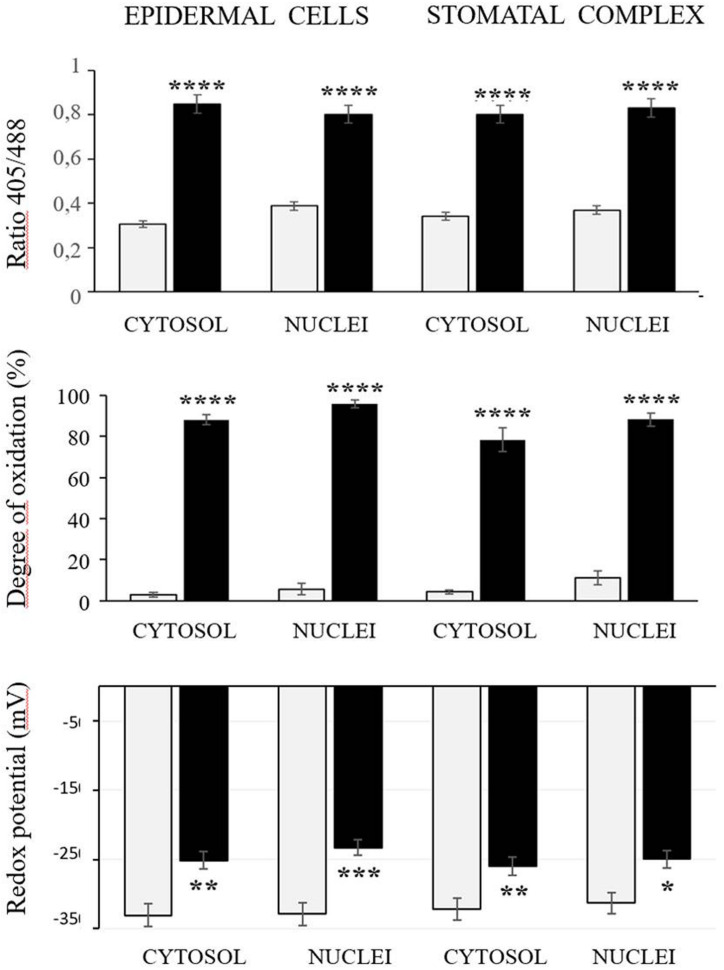
The 405/488 ratios, the degree of oxidation, and the glutathione redox potential in the cytosol and nuclei of the epidermal and stomatal guard cells of the control (white bars) and heat treated (black bars) seedlings. Asterisks indicate significant differences between control and heat-stressed plants according to the Student’s *t*-test (*p < 0.05; **p < 0.01; ***p < 0.001; and ****p < 0.0001).

The roGFP fluorescence was readily detected in the cells of the control and heat-treated leaves (Figure 4). Large changes in the 405/488 ratios of the cytosol and nuclei of the epidermal and stomatal guard cells were observed after the heat treatment (Figure 5). The degree of oxidation was low in the cytosol and nuclei of the epidermal and stomatal guard cells in the absence of HS (Figure 5), suggesting that the glutathione pool is highly reduced in these circumstances. However, the degree of oxidation was greatly increased in the nuclei and cytosol of both cell types following the HS treatment (Figure 5), demonstrating that HS caused substantial oxidation of the nuclei and cytosol. Similarly, the glutathione redox potentials of the cytosol and nuclei of the epidermal and stomatal guard cells were greatly changed as a result of HS (Figure 5).

**FIGURE 6:**
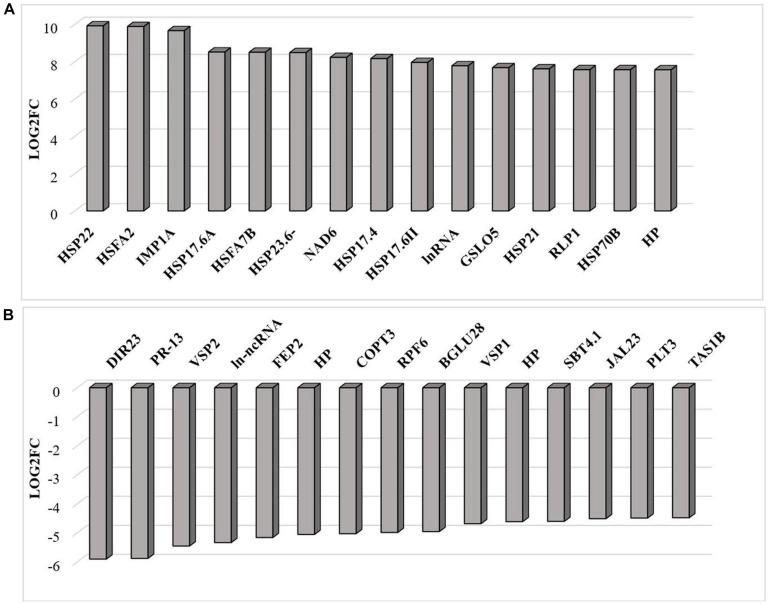
The transcripts that were most differentially changed in abundance after heat stress. The relative expression (log2) fold change-range of transcripts was annotated from their gene accession numbers from the TAIR website. Differentially expressed genes were those showing fold changes of (log2FC > 2 for up-regulated genes and log2FC < −2) for down-regulated genes, an FPKM > 1 and FDR-corrected *p-*value of 0.05 or less. DIR23 (AT2G2100), PR-13 (AT1G72260), VSP2 (AT5G24770), lncRNA (AT3G12502), FEP2 (AT1G47395), HP-hypothetical protein (AT3G06435), COPT3 (AT3G46900), RPF6 (AT1G63130), BGLU28 (AT2G44460), VSP1 (AT5G24780), HP-hypothetical protein (AT2G19970), SBT4.1 (AT5G59120), JAL23 (AT2G39330), PLT3 (AT2G18480), TAS1B (AT1G50055), HSP22 (AT4G10250), HSFA2 (AT2G26150), HSP17.6C (AT1G53540), HSP17.6A (AT5G12030), HSFA7B (AT3G63350), HSP23.6 (AT4G25200), HSP17.6A (AT5G12030), NAD6 (ATMG00270), HSP17.4A (AT3G46230), HSP17.6 (AT5G12020), natRNA (AT3G07365), CALS12 (AT5G03550), HSP21 (AT4G27670), RLP1 (AT1G07390), HSP17.8 (AT1G16030), HP-hypothetical protein (AT2G07779). **(A)** Increased in abundance and **(B)** decreased in abundance.

**FIGURE 7:**
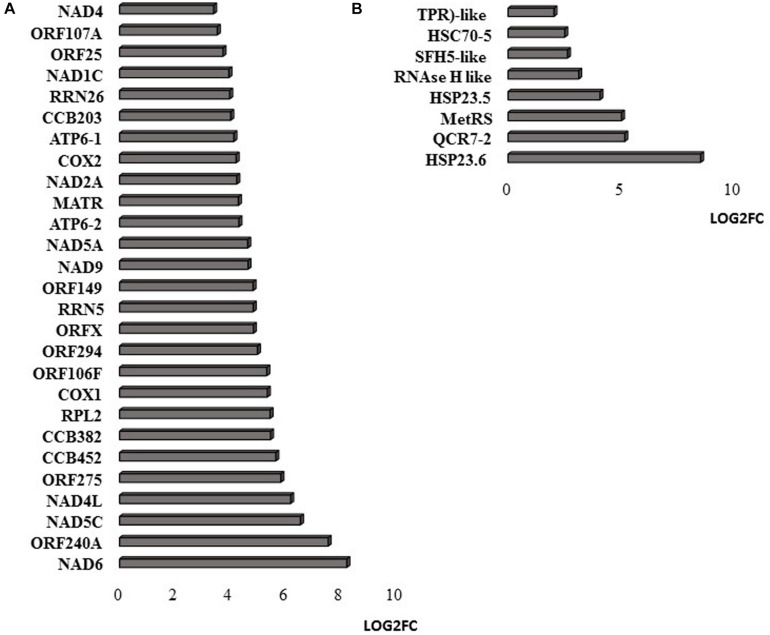
Mitochondrial-encoded transcripts **(A)** and nuclear-encoded transcripts targeted to mitochondria **(B)**. The abundance is expressed as relative expression of (log2) fold change (LOG2FC) with an FPKM > 1 and FDR-corrected *p-*value of 0.05 or less. NAD6 (ATMG00285), ORF240A (ATMG00382), NAD5C (ATMg00070), NAD4L (ATMG00660), ORF275 (ATMG00690), CCB452 (ATMG00270), CCB382 (ATMG00960), COX1 (ATMG01380), ORF106F (ATMG01170), ORF294 (ATMG01330), ORFX (ATMG00570), RRN5 (ATMG01390), ORF149 (ATMG00670), NAD9 (ATMG00090), NAD5A (ATMG00513), ATP6-2 (ATMG01200), MATR (ATMG00640), NAD2A (ATMG00285), COX2 (ATMG00180), ATP6-1 (ATMG00410), CCB203 (ATMG01130), RRN26 (ATMG00665), NAD1C (ATMG00520), ORF25 (ATMG00650), ORF107A (ATMG00030), NAD4 (ATMG00580), COX3 (ATMG00730), NAD7 (ATMG00510), ORF107H (ATMG01360), HSP23.6 (AT4G25200), QCR7-2 (AT5G25450), MetRS (AT5G10695), HSP23.5 (AT5G51440), RNAse H like (AT5G42965), SFH5-like (AT4G27580), HSC70-5 (AT5G09590), TPR-like (AT3G24000).

RNAseq analysis revealed that large numbers of transcripts associated with different mitochondrial functions were increased (Supplementary Figure 1). Other transcripts involved in plant defense, secretory pathways, and vacuolar functions were decreased in abundance (Supplementary Figure 2) in response to the HS. These trends can be clearly seen from the transcripts that were most abundant after HS (Figure 6A) and the transcripts that encode transcription factors and markers for phytohormone-dependent pathways that were significantly decreased in response to the HS (Figure 6B). In particular, transcripts encoding markers proteins such as the vegetative storage protein (VSP)1 and VSP2 for JA signaling pathways were decreased in abundance following exposure to HS (Hickman et al., 2017). The list of transcripts that were most highly expressed in response to HS includes those encoding the mitochondrially-localized HSP23.6, HSP70B proteins (Figure 6A), and HSP90.1 (Supplementary Figure 3). Moreover, transcripts that encode respiratory proteins and related mitochondrial functions were much less abundant in seedlings after exposure to HS (Figure 7). In addition to HS-induced decreases in transcripts encoding cytochrome C oxidase (COX) components (*COX1*, *COX2*) and a large number of components of the nicotinamide adenine dinucleotide [NAD(P)H] dehydrogenase complexes, transcripts involved in mitochondrial RNA editing and maturation were decreased in response to HS. Given the observed changes in transcripts that will ultimately limit mitochondria electron transport functions, it is surprising that exposure to HS was not found to elicit responses in mitochondrial retrograde responsive genes such as those encoding the mitochondrial alternative oxidases, particularly alternative oxidase 1a (Van Aken and Whelan, 2012).

**FIGURE 8:**
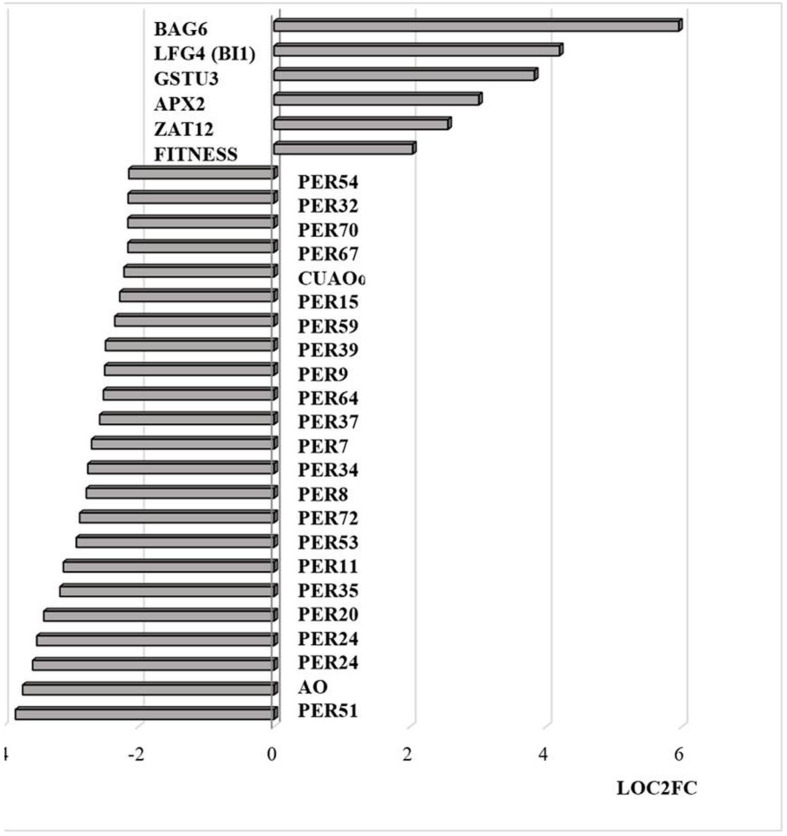
Transcripts encoding proteins associated with cell death and peroxidases that are differentially regulated by heat stress and targeted to the nucleus. The abundance is expressed as relative expression of (log2) fold change (LOG2FC) with an FPKM > 1 and FDR-corrected *p-*value of 0.05 or less. PER54 (AT5G06730), PER32 (AT3G32980), PER70 (AT5G64110), PER67 (AT5G58390), PER15 (AT2G18150), PER59 (AT5G19890), PER39 (AT4G11290), PER64 (AT5G42180), PER37 (AT4G08770), PER34 (AT3G49120), PER8 (AT1G34510), PER72 (AT5G66390), PER53 (AT5G06720), PER11 (AT1G68850), PER35 (AT3G49960), PER20 (AT2G35380), PER24 (AT2G39040), PER24 (AT2G39040), AO (AT5G21100), PER51 (AT4G37530), FITNESS (AT1G07050), ZAT12 (AT5G59820), APX2 (AT3G09640), GSTU3 (AT2G29470), LFG4(BI1) (AT1G03070), BAG6 (AT2G46240), PER7 (AT1G30870), CUAOα2 (AT1G31690), PER9 (AT1G34245).

**FIGURE 9:**
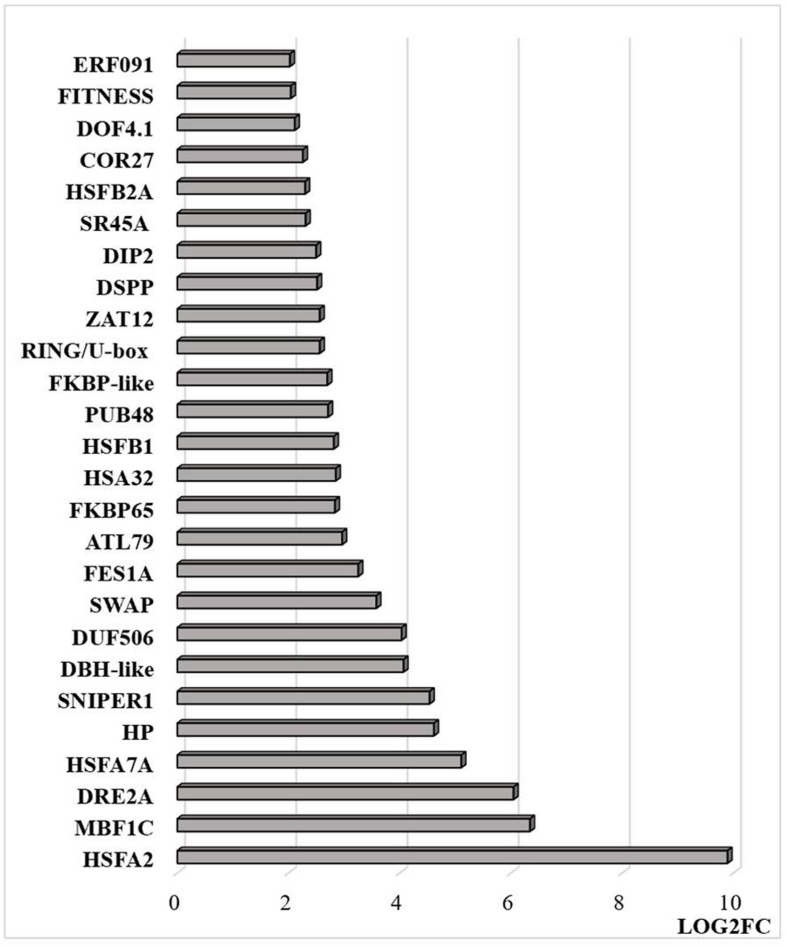
Transcripts that are differentially regulated by heat stress and targeted to the nucleus. The abundance is expressed as relative expression of (log2) fold change (LOG2FC) with an FPKM > 1 and FDR-corrected *p-*value of 0.05 or less. The thresholds were (LOG2FC > 2) and (LOG2FC < −2) for up- and down-regulated genes, respectively. HSFA2 (AT2G26150), MBF1C (AT4G25490), DRE2A (AT5G05410), HSFA7A (AT3G51910), HP—hypothetical protein (At1g21550), SNIPER1 (AT1G14200), DBH-like (AT5G35320), DUF506 (AT3G25240), SWAP (AT3G49130), FES1A (AT3G09350), ATL79 (AT5G47610), FKBP65 (AT5G48570), HSA32 (AT4G21320), HSFB1 (AT4G36990), PUB48(AT5G18340), FKBP-like (AT5G03990), RING/U-box (AT5G05530), ZAT12 (AT5G59820), DSPP (AT1G07330), DIP2 (AT5G03210), SR45A (AT1G07350), HSFB2A (AT5G62020), COR27 (AT5G42900), DOF4.1 (AT4G00940), FITNESS (AT1G07050), ERF091 (AT4G18450).

Similar to transcripts encoding APX2, transcripts encoding the Zinc finger protein ZAT12, which is involved in oxidative stress signaling (Nguyen et al., 2012), were highly expressed in response to HS (Figure 8). It has previously been shown that HSFA4A binds to the promoters of transcription factors such as WRKY30 and ZAT12 leading to enhanced tolerance to heat through reduction of oxidative damage (Andrási et al., 2019). Exposure to HS also increased the levels of transcripts encoding glutathione transferase (GST) U3 and LIFEGUARD4 (LF4), which is a BCL2 associated X, apoptosis regulator (BAX)-inhibitor1 family protein (Figure 8). Exposure to HS results in the movement of the glycolytic enzyme cytosolic glyceraldehyde-3-phosphate dehydrogenase (GAPC) to the nucleus, where it mediates HS responses through association with NF-YC10 (Kim et al., 2020). Overexpression of GAPC1 enhanced the expression of a HS genes including LF4 (Kim et al., 2020). The nuclear localized calmodulin binding protein Bcl-2 associated athanogene (BAG)6, which is a regulator of programmed cell death, was highly expressed in response the HS (Figure 8). BAG6 is a HSP70-binding protein that suppresses the expression of Fes1A and plays a positive role in thermotolerance in Arabidopsis (Fu et al., 2019). However, the levels of *Fes1A* transcripts were found to be more abundant after HS treatment in the current experiments (Figure 9). The heat-induced expression of *HSP18.2* and *HSP25.3* in *bag6* mutants was found to correlate with enhanced thermotolerance, suggesting that BAG6 restricts the induction of some sHSPs, limiting the extension of the HS response through regulation of the transcriptional cascade (Echevarría-Zomeño et al., 2016). In contrast to the above genes, transcripts encoding large numbers of apoplastic peroxidases and cell wall associated ascorbate oxidases (AOs) were less abundant after exposure to HS (Figure 8).

**FIGURE 10:**
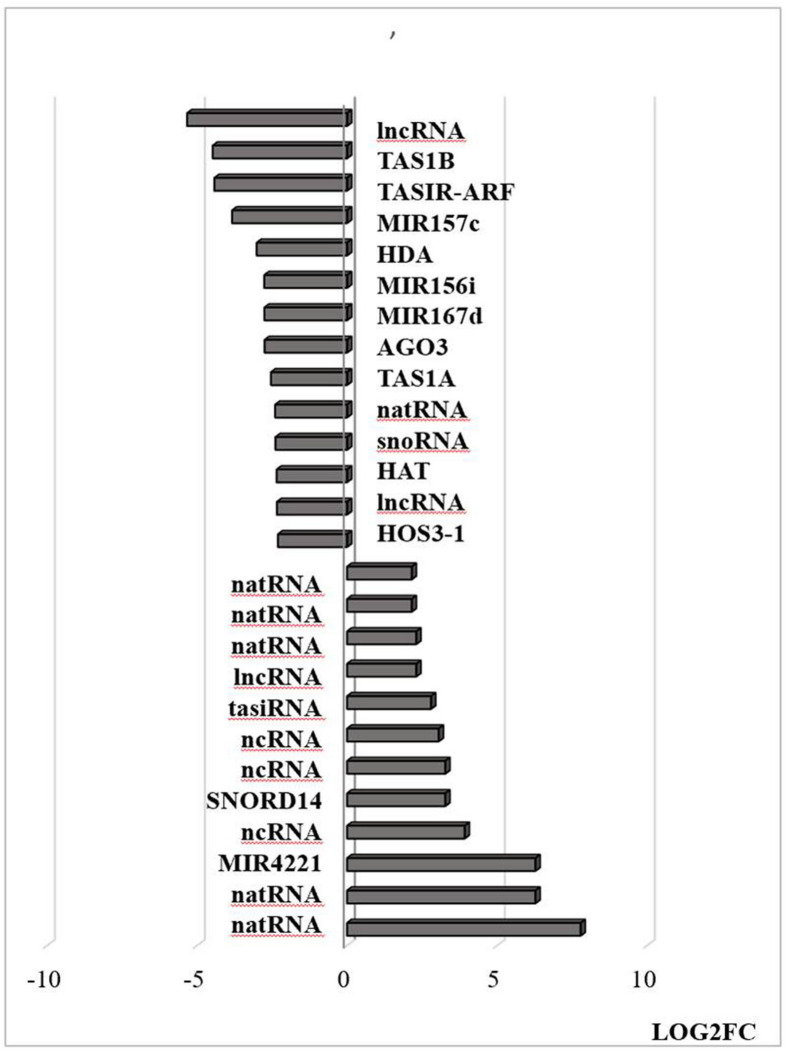
Transcripts associated with small RNAs that are differentially regulated by heat stress. Examples of this class include Argonaute complexes (AGO) and histone deacetylase (HDAC). The abundance is expressed as (log2) fold change (LOG2FC). natural antisense RNAs (AT3G07365, AT3G04685, AT3G04685, AT1G04767, AT1G04767, AT5G03185); trans-acting siRNAs [AT1G63150, AT1G31130 (TAS1A), AT5G57735 (TASIR-ARF), AT1G50055 (TAS1B); AT1G75166 (snoRNA)], non-coding RNA (AT3G03215, AT3G41761, AT2G05205, AT4G06265, AT3G05655, AT5G03185, AT3G12502); microRNAs [AT4G06260 (MIR4221), AT1G31173 (MIR167D), AT1G07867 (MIR167D), AT1G31173 (MIR156i), AT3G18217 (MIR157C)] and proteins; AT1G31290 (AGO3), AT1G24145 (HDAC).

Transcripts encoding transcription factors and related proteins that are localized in the nucleus were more abundant after HS treatment (Figure 9). In particular, transcripts encoding HSFA2, HASFA7, FKBP62 (ROF1, that interacts with HSP90.1 and modulates HsfA2), and the transcriptional co-activator multiprotein bridging factor 1 (MBF)1c were greatly increased in abundance after the HS treatment. These proteins are key regulators of thermotolerance in Arabidopsis. For example, MBF1c is a transcriptional regulator of HS response genes, including the DRE-binding protein 2A (DREB2A), HSFs, and zinc finger proteins. In addition, the levels of transcripts encoding the E3 ligases SNIPER1, which modulates plant immune responses through ubiquitination, and PUB48 that regulates plant abiotic stress responses, were more abundant after HS treatment (Figure 9). In addition, exposure to HS resulted in marked changes in the levels of transcripts encoding components that are involved in the epigenetic regulation of gene expression (Figure 10).

## Discussion

The exposure to heat triggers a general accumulation of ROS in plant organs, together with the activation (or inactivation) of multiple redox-regulated proteins that often contain thiol-disulfide switches. HS-induced ROS production and associated redox signaling are intimately associated with the HS responses that underpin thermotolerance, particularly the expression of HSFs and HSPs (Driedonks et al., 2015). Heat directly and indirectly (via ROS) stimulates HSF activity. In turn, HSFs stimulate the expression of HSP chaperones and ROS-processing proteins and antioxidant-related transcription factors such as ZAT10 and HSFA1D, which stimulates the *APX2* expression. The HS-induced changes in the transcriptome profile reported here are similar to previous reports, with the increase in transcripts encoding HSPs and significant downregulation of membrane proteins such as transporters (Poidevin et al., 2020).

**FIGURE 11:**
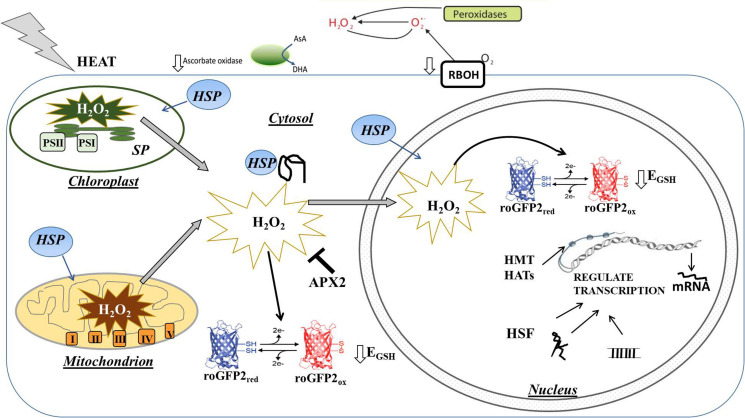
Simplified model illustrating the effect of heat stress on H_2_O_2_ production and associated oxidation of the cytosol and nucleus on the regulation of gene expression. The levels of transcripts encoding apoplastic H_2_O_2_ producing enzymes such as the Respiratory Burst Homologs (RBOH) and cell wall peroxidases were decreased in response to heat, as were transcripts encoding ascorbate oxidase (AO), which catalyzes the first step of the ascorbate degradation pathway. Heat increases the production of H_2_O_2_ in chloroplasts and mitchondria leading to oxidation of the cytosol and nucleus. This oxidation causes a highly specific expression of antioxidant genes such as APX2 that are important in the regulation of signal transduction, as well as heat shock factors (HSF) that regulate heat shock-induced signaling process. Transcription is also rapidly regulated by epigenetic factors such as histone methyltransferases (HMT) and histone acetyltransferases (HAT) as well as by the production of microRNAs (miRNA) and long non-coding RNA (lncRNA).

While HS is likely to trigger different levels of ROS accumulation in each intracellular and inter(extra)cellular compartment, relatively little information is available on the compartment-specific redox changes that are induced in response to HS. Although the exact mechanisms by which the separate redox pools in different compartments interact and orchestrate cell signaling remain unclear, oxidation in each cellular compartment is likely to transmit specific signals that facilitate an appropriate change in gene expression (Noctor and Foyer, 2016). For example, H_2_O_2_ produced in the apoplast/cell wall can be directly sensed by membrane receptor kinases such as HPCA1 (also called CANNOT RESPOND TO DMBQ1; CARD1), which triggers an influx of calcium ions into the cell, leading to the activation of MAP kinases and other signaling pathways (Wu et al., 2020). The data presented here show that the levels of transcripts encoding apoplastic H_2_O_2_-producing enzymes such as the Respiratory Burst Homologs (RBOH) and cell wall peroxidases are decreased in response to heat, transcripts encoding AO. The heat-induced activation of the respiratory burst oxidase homolog 1 (RBOH1) was reported to be important in the apoplastic oxidative burst that triggers MAP kinase signaling cascades leading to thermotolerance (Zhou et al., 2014). However, the observed changes in transcripts encoding cell wall peroxidases and AO reported here would favor decreased apoplastic H_2_O_2_ production and a more reduced state of the apoplast, as illustrated in Figure 11. The decrease in AO transcripts may lead to a higher level of ascorbate in the apoplast and ultimately influence the overall process of acclimation to HS, in a similar manner to the role of AO in acclimation to high light (Karpinska et al., 2017a).

Like mitochondria, chloroplasts play an important role in heat-induced ROS accumulation and in the resultant expression of nuclear heat-response genes (Hu et al., 2020). The data presented here demonstrate that the cytosol and nuclei become highly oxidized in response to HS-induced ROS production. Relatively, little attention has been paid to how direct oxidation will influence nuclear proteins and their roles in the acclimation process (Martins et al., 2018). The high temperature-induced accumulation of ROS is often discussed in terms of oxidative stress, cell injury, and death (Nishad and Nandi, 2020). However, ROS production is essential for plant growth and development (Considine et al., 2017; Mittler, 2017) and ROS accumulation is required for the activation of Hsfs and thermotolerance (Hasanuzzaman et al., 2013; Rezaei et al., 2015). The HS transcriptome signature reported here demonstrates that relatively transcripts associated with antioxidant status are increased in abundance, suggesting that there is no generic antioxidant response to counteract the HS-induced oxidation. In contrast, the only antioxidant transcript that was increased is APX2, suggesting a very specific redox-processing response to HS. In addition, transcripts encoding ZAT12 were increased in the seedlings after HS, suggesting altered oxidative stress signaling (Nguyen et al., 2012).

The nucleus also contains glutathione, glutaredoxins, thioredoxins, and thiols reductases to process ROS, as well as proteins with redox-regulated cysteines that regulate nuclear functions, such as gene expression, transcription, epigenetics, and chromatin remodeling (Martins et al., 2018). The oxidation of redox-regulated transcription factors in the nucleus will have a direct influence on gene expression (He et al., 2018). For example, the AP2/ethylene response factor (ERF) transcription factors are subject to redox regulation (Vogel et al., 2014) as are the ROXY proteins that interact with TGA transcription factors (Dietz, 2014; Delorme-Hinoux et al., 2016). The heat-induced oxidation of the nucleus observed in the present study will undoubtedly regulate the functions of these redox-sensitive transcription factors (Rouhier et al., 2015; Waszczak et al., 2015). Oxidation of the cytosol can also trigger the movement of redox-sensitive proteins into the nucleus (Foyer et al., 2020). In particular, the oxidative of the cytosol triggers the movement of GAPC to the nucleus, where it associates with NF-YC10 to mediate HS responses (Kim et al., 2020). Other examples of oxidation-induced protein translocation to the nucleus include HSFA1D and HSFA8 (Giesguth et al., 2015; Dickinson et al., 2018) and the NON-EXPRESSOR OF PR GENES1 (NPR1), which is an important component of the SA-dependent transcriptional response (Kneeshaw et al., 2014).

In addition, the epigenome is re-modeled by heat-induced priming (Liu et al., 2015). Many of the enzymes involved in histone methylation are subject to redox regulation, which affects both positive and negative histone marks (e.g., H3K4me2, H3K4me3, H3K79me3, H3K27me2, and H3K9me2) which control recombination in meiosis and other processes (Niu et al., 2015). Histone acetylation is regulated by redox changes in the nucleus of mammals, to alter chromatin conformation and transcription (Doyle and Fitzpatrick, 2010). While this type of regulation has not yet been described in plants, reactive nitrogen species such as nitric oxide (NO) induce inhibition of histone deacetylases (HDACs) leading to the stress-induced regulation of gene transcription (Mengel et al., 2017). Histone H3 is glutathionylated in mammals on a conserved and unique Cys residue. The level of H3 glutathionylation increases during cell proliferation and aging, leading to a more open chromatin structure (García-Giménez et al., 2013, 2014, 2017). Moreover, members of the Arabidopsis DICER-LIKE (DCL) and RNASE THREE-LIKE (RTL) endonucleases families are glutathionylated on a conserved Cys, a process that changes their RNase III activities (Charbonnel et al., 2017). Therefore, the synthesis of small RNA and their subsequent regulation of gene expression are under the control of the cellular redox environment (Charbonnel et al., 2017).

DNA methylation is a major mechanism of epigenetic regulation of gene expression in plants. Redox regulation of the enzymes of the S-adenosyl methionine (SAM) cycle, which provide precursors for DNA and histone methylation, may be important in the regulation of these processes. Other likely targets for redox regulation are the DNA demethylases called Repressor of Silencing 1 (ROS1) and the Demeter-like (DME, DML2, and DML3) enzymes, which remove methylated bases from the DNA backbone (Zhu, 2009). Moreover, the cytosolic Fe-S cluster assembly enzymes such as MET18 and AE7 that are involved in DNA methylation might be subject to redox regulation because they affect nuclear DNA demethylases Fe-S cluster metabolism. In addition, miRNA-mediated epigenetic changes constitute an additional regulatory mechanism of activating HS responses. HS-regulated miRNAs and their target genes that are associated with thermotolerance were recently characterized in wheat (Ravichandran et al., 2019). The target genes of miRNA156, miR159, miR166, and miR398 were shown to be conserved between species such as wheat, other cereals, and dicotyledonous plants (Ravichandran et al., 2019). Chloroplast and mitochondria localized pentatricopeptide repeat-containing and mitochondrial transcription termination factor-like proteins were regulated through miRNA-guided cleavage (Ravichandran et al., 2019).

## Conclusion

The data presented here shed new light on the intracellular compartmentation of heat-induced redox changes with plant cells and highlight the significant oxidation of the nucleus as well as the cytosol following HS, as illustrated in Figure 11. Few studies to date have described direct stress-induced oxidation of the nucleus and considered how the resultant oxidation of the nuclear proteins influences the observed genetic and epigenetic changes. Rather than regarding the accumulation of ROS as essentially a harmful consequence of HS, the data presented here suggest that specific redox processing and signaling pathways are triggered, as evidenced by the differential transcriptional responses in the APX and peroxidase genes. The compartment-specific increases in oxidation, together with the relative changes in redox state between the different cellular compartments are crucial to the signaling that underpins the HS response. Moreover, considerations of the oxidative regulation of nuclear proteins may identify important mechanisms of epigenetic regulation of thermotolerance.

## Data Availability Statement

The datasets presented in this study can be found in online repositories. The names of the repository/repositories and accession number(s) can be found below: NCBI, accession nos: PRJNA669354 and SRP287927.

## Author Contributions

RB, BK, AG, and CF planned the experiments. RB and BK undertook the experimental work and data analysis. BK produced the figures. CF co-wrote the manuscript. All authors contributed to the article and approved the submitted version.

## Conflict of Interest

The authors declare that the research was conducted in the absence of any commercial or financial relationships that could be construed as a potential conflict of interest.

## Footnotes

1 http://rsbweb.nih.gov/ij/

## Supplementary Material

Supplementary Figure 1 Pie diagrams of transcripts showing heat-induced increases in abundance according to GO enrichment. All RNAs were analyzed for their respective Gene Ontology (GO) terms and fold enrichment through the GO consortium and PANTHER classification system, using *Arabidopsis thaliana* as a reference genome. GO enrichment that is over-represented in up-regulated set of transcripts was analyzed using to the PANTHER Classification System which contains up-to-date GO annotation data for Arabidopsis and other plant species (https://arabidopsis.org/tools/go_term_enrichment.jsp). Pie diagrams represent GO-terms for biological processes, molecular functions, and cellular components.

Supplementary Figure 2 Pie diagrams of transcripts showing heat-induced decreases in abundance according to GO enrichment for biological processes, molecular functions, and cellular components. All RNAs were analyzed for their respective gene ontology (GO) terms and fold enrichment through the GO consortium and PANTHER classification system, using *Arabidopsis thaliana* as a reference genome. GO enrichment that is over-represented in up-regulated set of transcripts was analyzed using to the PANTHER Classification System which contains up-to-date GO annotation data for Arabidopsis and other plant species (https://arabidopsis.org/tools/go_term_enrichment.jsp). Pie diagrams represent GO-terms for biological processes, molecular functions, and cellular components.

Supplementary Figure 3 Transcripts encoding heat shock proteins and heat shock factors that are differentially changed in abundance in response to heat stress.

Supplementary Table 1 RNAseq data used for the analysis of heat stress responses.

## References

[B1] Ahamed K. U. Nahar K. Fujita M. Hasanuzzaman M. (2010). Variation in plant growth, tiller dynamics and yield components of wheat (*Triticum aestivum* L.) due to high temperature stress. *Adv. Agric. Bot.* 2 213–224.

[B2] Andrási N. Rigó G. Zsigmond L. Pérez-Salamó I. Papdi C. Klement E. (2019). The mitogen-activated protein kinase 4-phosphorylated heat shock factor A4A regulates responses to combined salt and heat stresses. *J. Exp. Bot.* 70 4903–4917. 10.1093/jxb/erz217 31086987 PMC6760271

[B3] Balfagón D. Sengupta S. Gómez-Cadenas A. fritschi F. B. Azad R. Mittler R. (2019). Jasmonic acid is required for plant acclimation to a combination of high light and heat stress. *Plant Physiol.* 181 1668–1682. 10.1104/pp.19.00956 31594842 PMC6878009

[B4] Banti V. Mafessoni F. Loreti E. Alpi A. Perata P. (2010). The heat-inducible transcription factor *HsfA2* enhances anoxia tolerance in Arabidopsis. *Plant Physiol.* 152 1471–1483. 10.1104/pp.109.149815 20089772 PMC2832282

[B5] Baxter A. Mittler R. Suzuki N. (2014). ROS as key players in plant stress signalling. *J. Exp. Bot.* 65 1229–1240. 10.1093/jxb/ert375 24253197

[B6] Berry J. Bjorkman O. (1980). Photosynthetic response and adaptation to temperature in higher plants. *Annu. Rev. Plant Physiol.* 31 491–543. 10.1146/annurev.pp.31.060180.002423

[B7] Charbonnel C. Niazi A. K. Elvira-Matelot E. Nowak E. Zytnicki M. de Bures A. (2017). The siRNA suppressor RTL1 is redox-regulated through glutathionylation of a conserved cysteine in the double-stranded-RNA-binding domain. *Nucleic Acids Res.* 45 11891–11907. 10.1093/nar/gkx820 28981840 PMC5714217

[B8] Choudhury F. K. Rivero R. M. Blumwald E. Mittler R. (2017). Reactive oxygen species, abiotic stress and stress combination. *Plant J.* 90 856–867. 10.1111/tpj.13299 27801967

[B9] Considine M. Diaz Vivancos P. Kerchev P. Signorelli S. Agudelo-Romero P. Gibbs D. J. (2017). Learning to breathe: developmental phase transitions in oxygen status. *Trends Plant Sci.* 22 140–153. 10.1016/j.tplants.2016.11.013 27986423

[B10] de Simone A. Hubbard R. de la Torre N. V. Velappan Y. Wilson M. Considine M. J. (2017). Redox changes during the cell cycle in the embryonic root meristem of *Arabidopsis thaliana*. *Antioxid. Redox Signal.* 27 1505–1519. 10.1089/ars.2016.6959 28457165 PMC5678362

[B11] Delorme-Hinoux V. Bangash S. A. K. Meyer A. J. Reichheld J.-P. (2016). Nuclear thiol redox systems in plants. *Plant Sci.* 243 84–95. 10.1016/j.plantsci.2015.12.002 26795153

[B12] Dickinson P. J. Kumar M. Martinho C. Yoo S. J. Lan H. Artavanis G. (2018). Chloroplast signalling gates thermotolerance in *Arabidopsis*. *Cell Rep.* 22 1657–1665. 10.1016/j.celrep.2018.01.054 29444421 PMC5847188

[B13] Dietz K.-J. (2014). Redox regulation of transcription factors in plant stress acclimation and development. *Antioxid. Redox Signal.* 21 1356–1372. 10.1089/ars.2013.5672 24182193

[B14] Dietz K.-J. Turkan I. Krieger-Liszkay A. (2016). Redox- and reactive oxygen species- dependent signaling into and out of the photosynthesizing chloroplast. *Plant Phys.* 171 1541–1550. 10.1104/pp.16.00375 27255485 PMC4936569

[B15] Doyle K. Fitzpatrick F. A. (2010). Redox signaling, alkylation (carbonylation) of conserved cysteines inactivates class I histone deacetylases 1, 2, and 3 and antagonizes their transcriptional repressor function. *J. Biol. Chem.* 285 17417–17424. 10.1074/jbc.m109.089250 20385560 PMC2878505

[B16] Driedonks N. Xu J. Peters J. L. Park S. Rieu I. (2015). Multi-level interactions between heat shock factors, heat shock proteins, and the redox system regulate acclimation to heat. *Front. Plant Sci.* 6:999. 10.3389/fpls.2015.00999 26635827 PMC4647109

[B17] Easterling D. R. Apps M. (2005). Assessing the consequences of climate change for food and forest resources: a view from the IPCC. *Clim. Change* 70 165–180. 10.1007/1-4020-4166-7_8

[B18] Echevarría-Zomeño S. Fernández-Calvino L. Castro-Sanz A. B. López J. A. Vázquez J. Mar Castellano M. (2016). Dissecting the proteome dynamics of the early heat stress response leading to plant survival or death in *Arabidopsis*. *Plant Cell Environ.* 39 1264–1278. 10.1111/pce.12664 26580143

[B19] Foyer C. H. Baker A. Wright M. Sparkes I. Mhamdi A. Schippers J. H. M. (2020). On the move: redox –dependent protein relocation. *J. Exp. Bot.* 71 620–631. 10.1093/jxb/erz330 31421053

[B20] Fu C. Hou Y. Ge J. Zhang L. Liu X. Huo P. (2019). Increased *fes1a* thermotolerance is induced by BAG6 knockout. *Plant Mol. Biol.* 100 73–82. 10.1007/s11103-019-00844-8 30796711

[B21] García-Giménez J. L. Ibañez-Cabellos J. S. Seco-Cervera M. Pallardó F. V. (2014). Glutathione and cellular redox control in epigenetic regulation. *Free Radic. Biol. Med.* 75:S3. 10.1016/j.freeradbiomed.2014.10.828 26461333

[B22] García-Giménez J. L. Òlaso G. Hake S. B. Bönisch C. Wiedemann S. M. Markovic J. (2013). Histone h3 glutathionylation in proliferating mammalian cells destabilizes nucleosomal structure. *Antioxid. Redox Signal.* 19 1305–1320. 10.1089/ars.2012.5021 23541030 PMC3791047

[B23] García-Giménez J. L. Romá-Mateo C. Pérez-Machado G. Peiró-Chova L. Pallardó F. V. (2017). Role of glutathione in the regulation of epigenetic mechanisms in disease. *Free Radic. Biol. Med.* 112 36–48. 10.1016/j.freeradbiomed.2017.07.008 28705657

[B24] García-Quirós E. de Dios Alché J. Karpinska B. Foyer C. H. (2020). Glutathione redox state plays a key role in flower development and pollen vigour. *J. Exp. Bot.* 71 730–741. 10.1093/jxb/erz376 31557297 PMC6946011

[B25] Giesguth M. Sahm A. Simon S. Dietz K.-J. (2015). Redox-dependent translocation of the heat shock transcription factor AtHSFA8 from the cytosol to the nucleus in *Arabidopsis thaliana*. *FEBS Lett.* 589 718–725. 10.1016/j.febslet.2015.01.039 25666709

[B26] Giorno F. Wolters-Arts M. Mariani C. Rieu I. (2013). Ensuring reproduction at high temperatures: the heat stress response during anther and pollen development. *Plants* 2 489–506. 10.3390/plants2030489 27137389 PMC4844380

[B27] Groten K. Vanacker H. Dutilleul C. Bastian F. Bernard S. Carzaniga R. (2005). The roles of redox processes in pea nodule development and senescence. *Plant Cell Environ.* 28 1293–1304. 10.1111/j.1365-3040.2005.01376.x

[B28] Hanson G. T. Aggeler R. Oglesbee D. Cannon M. Capaldi R. A. Tsien R. Y. (2004). Investigating mitochondrial redox potential with redox-sensitive green fluorescent protein indicators. *J. Biol. Chem.* 279 13044–13053. 10.1074/jbc.m312846200 14722062

[B29] Hasanuzzaman M. Nahar K. Alam M. M. Roychowdhury R. Fujita M. (2013). Physiological, biochemical, and molecular mechanisms of heat stress tolerance in plants. *Int. J. Mol. Sci.* 14 9643–9684. 10.3390/ijms14059643 23644891 PMC3676804

[B30] He H. Van Breusegem F. Mhamdi F. (2018). Redox-dependent control of nuclear transcription in plants. *J. Exp. Bot.* 69 3359–3372. 10.1093/jxb/ery130 29659979

[B31] Hickman R. Van Verk M. C. Van Dijken A. J. H. Pereira Mendes M. Vroegop-Vos I. A. Caarls L. (2017). Architecture and dynamics of the jasmonic acid gene regulatory network. *Plant Cell* 29 2086–2105. 10.1105/tpc.16.00958 28827376 PMC5635973

[B32] Hu S. Ding Y. Zhu C. (2020). Sensitivity and responses of chloroplasts to heat stress in plants. *Front. Plant Sci.* 11:375 10.3389/fpls.2020.00375 PMC7142257 32300353

[B33] Huang J. Zhao X. Chory J. (2019). The *Arabidopsis* transcriptome responds specifically and dynamically to high light stress. *Cell Rep.* 29 4186–4199.e3. 31851942 10.1016/j.celrep.2019.11.051 PMC7030938

[B34] Karpinska B. Owdah Alomrani S. Foyer C. H. (2017a). Inhibitor-induced oxidation of the nucleus and cytosol in *Arabidopsis thaliana*: implications for organelle to nucleus retrograde signalling. *Philos. Trans. R. Soc. B Biol. Sci.* 372:20160392. 10.1098/rstb.2016.0392 28808105 PMC5566886

[B35] Karpinska B. Rasool B. Zhang K. Pastok D. Morris J. Verrall S. R. (2017b). The redox state of the apoplast influences the acclimation of photosynthesis and leaf metabolism to changing irradiance. *Plant Cell Environ.* 41 1083–1097. 10.1111/pce.12960 28369975 PMC5947596

[B36] Kim S.-C. Guo L. Wang X. (2020). Nuclear moonlighting of cytosolic glyceraldehyde 3-phosphate dehydrogenase regulates Arabidopsis response to heat stress. *Nat. Commun.* 11:3439. 10.1038/s41467-020-17311-4 PMC7351759 32651385

[B37] Kneeshaw S. Gelineau S. Tada Y. Loake G. J. Spoel S. H. (2014). Selective protein denitrosylation activity of Thioredoxin-h5 modulates plant immunity. *Mol. Cell* 56 153–162. 10.1016/j.molcel.2014.08.003 25201412

[B38] Liu H. C. Charng Y. Y. (2013). Common and distinct functions of Arabidopsis class A1 and A2 heat shock factors in diverse abiotic stress responses and development. *Plant Physiol.* 163 276–290. 10.1104/pp.113.221168 23832625 PMC3762648

[B39] Liu J. Feng L. Li J. He Z. (2015). Genetic and epigenetic control of plant heat responses. *Front. Plant Sci.* 6:267. 10.3389/fpls.2015.00267 25964789 PMC4408840

[B40] Martins L. Trujillo-Hernandez J. A. Reichheld J.-P. (2018). Thiol based redox signaling in plant nucleus. *Front. Plant Sci.* 9:705. 10.3389/fpls.2018.00705 29892308 PMC5985474

[B41] Meiri D. Breiman A. (2009). Arabidopsis ROF1 (FKBP62) modulates thermotolerance by interacting with HSP90.1 and affecting the accumulation of HsfA2-regulated sHSPs. *Plant J.* 59 387–399. 10.1111/j.1365-313x.2009.03878.x 19366428

[B42] Mengel A. Ageeva A. Georgii E. Bernhardt J. Wu K. Durner J. (2017). Nitric oxide modulates histone acetylation at stress genes by inhibition of histone deacetylases. *Plant Physiol.* 173 1434–1452. 10.1104/pp.16.01734 27980017 PMC5291017

[B43] Meyer A. J. Brach T. Marty L. Kreye S. Rouhier N. Jacquot J. P. (2007). Redox-sensitive GFP in *Arabidopsis thaliana* is a quantitative biosensor for the redox potential of the cellular glutathione redox buffer. *Plant J.* 52 973–986. 10.1111/j.1365-313x.2007.03280.x 17892447

[B44] Mittler R. (2017). ROS are good. *Trends Plant Sci.* 22 11–19. 10.1016/j.tplants.2016.08.002 27666517

[B45] Nguyen X. C. Kim S. H. Lee K. (2012). Identification of a C2H2-type zinc finger transcription factor (ZAT10) from *Arabidopsis* as a substrate of MAP kinase. *Plant Cell Rep.* 31 737–745. 10.1007/s00299-011-1192-x 22134874

[B46] Nietzel T. Elsässer M. Ruberti C. Steinbeck J. Ugalde J. M. Fuchs P. (2019). The fluorescent protein sensor roGFP2-Orp1 monitors *in vivo* H2O2 and thiol redox integration and elucidates intracellular H2O2 dynamics during elicitor-induced oxidative burst in Arabidopsis. *New Phytol.* 221 1649–1664. 10.1111/nph.15550 30347449

[B47] Niinemets Ü. (2018). When leaves go over the thermal edge. *Plant Cell Environ.* 41 1247–1250. 10.1111/pce.13184 29508926

[B48] Nishad A. Nandi A. K. (2020). Recent advances in plant thermomemory. *Plant Cell Rep.* 10.1007/s00299-020-02604-1 [Epub ahead of print]. 32975635

[B49] Niu Y. DesMarais T. L. Tong Z. Yao Y. Costa M. (2015). Oxidative stress alters global histone modification and DNA methylation. *Free Radic. Biol. Med.* 82 22–28. 10.1016/j.freeradbiomed.2015.01.028 25656994 PMC4464695

[B50] Noctor G. Foyer C. H. (2016). Intracellular redox compartmentation and ROS-related communication in regulation and signalling. *Plant Physiol.* 171 1582–1592. 10.1104/pp.16.00346 PMC4936564 27208308

[B51] Noctor G. Mhamdi A. Foyer C. H. (2016). Oxidative stress and antioxidative systems: recipes for successful data collection and interpretation. *Plant Cell Environ.* 39 1140–1160. 10.1111/pce.12726 26864619

[B52] Noctor G. Queval G. Mhamdi A. Chaouch S. Foyer C. H. (2011). “Glutathione,” in *The Arabidopsis Book*, Vol. 9 ed. Millar H. (Rockville, MD: American Society of Plant Biologists), 1–32. 10.1199/tab.0142 PMC3267239 22303267

[B53] Noctor G. Riechheld J.-P. Foyer C. H. (2018). ROS-related redox regulation and signaling in plants. *Semin. Cell Dev. Biol.* 80 3–12. 10.1016/j.semcdb.2017.07.013 28733165

[B54] Ogawa D. Yamaguchi K. Nishiuchi T. (2007). High-level overexpression of the *Arabidopsis HsfA2* gene confers not only increased themotolerance but also salt/osmotic stress tolerance and enhanced callus growth. *J. Exp. Bot.* 58 3373–3383. 10.1093/jxb/erm184 17890230

[B55] Perdomo J. A. Capó-Bauçà S. Carmo-Silva E. Galmés J. (2017). Rubisco and rubisco activase play an important role in the biochemical limitations of photosynthesis in rice, wheat, and maize under high temperature and water deficit. *Front. Plant Sci.* 8:490. 10.3389/fpls.2017.00490 28450871 PMC5390490

[B56] Piramila B. H. M. Prabha A. L. Nandagopalan V. Stanley A. L. (2012). Effect of heat treatment on germination, seedling growth and some biochemical parameters of dry seeds of black gram. *Int. J. Pharm. Phytopharmacol. Res.* 1 194–202.

[B57] Poidevin L. Forment J. Unal D. Ferrando A. (2020). Transcriptome and translatome changes in germinated pollen under heat stress uncover roles of transporter genes involved in pollen tube growth. *BioRXiv [Preprint]* 10.1101/2020.05.29.122937 33289138

[B58] Portis A. R. Jr. (2003). Rubisco activase – Rubisco’s catalytic chaperone. *Photosynth. Res.* 75 11–27. 16245090 10.1023/A:1022458108678

[B59] Ravichandran S. Ragupathy R. Edwards T. Domaratzki M. Cloutier S. (2019). MicroRNA-guided regulation of heat stress response in wheat. *BMC Genomics* 20:488. 10.1186/s12864-019-5799-6 31195958 PMC6567507

[B60] Rezaei E. E. Webber H. Gaiser T. Naab J. Ewert F. (2015). Heat stress in cereals: mechanisms and modelling. *Eur. J. Agron.* 64 98–113. 10.1016/j.eja.2014.10.003

[B61] Rossi S. Burgess P. Jespersen D. Huang B. (2017). Heat-induced leaf senescence associated with chlorophyll metabolism in bentgrass lines in heat tolerance. *Crop Sci.* 57 169–178.

[B62] Rouhier N. Cerveau D. Couturier J. Reichheld J.-P. Rey P. (2015). Involvement of thiol-based mechanisms in plant development. *Biochim. Biophys. Acta* 1850 1479–1496. 10.1016/j.bbagen.2015.01.023 25676896

[B63] Sage R. F. Way D. A. Kubien D. S. (2008). Rubisco, rubisco activase, and global climate change. *J. Exp. Bot.* 59 1581–1595. 10.1093/jxb/ern053 18436544

[B64] Sato S. Kamiyama M. Iwata T. Makita N. Furukawa H. Ikeda H. (2006). Moderate increase of mean daily temperature adversely affects fruit set of *Lycopersicon esculentum* by disrupting specific physiological processes in male reproductive development. *Ann. Bot.* 97 731–738. 10.1093/aob/mcl037 16497700 PMC2803419

[B65] Schnaubelt D. Dong Y. Queval G. Diaz-Vivancos P. Makgopa M. E. Howell G. (2015). Low glutathione regulates gene expression and the redox potentials of the nucleus and cytosol in *Arabidopsis thaliana*. *Plant Cell Environ.* 38 266–279. 10.1111/pce.12252 24329757

[B66] Schramm F. Ganguli A. Kiehlmann E. Englich G. Walch D. von Koskull-Doring P. (2006). The heat stress transcription factor HsfA2 serves as a regulatory amplifier of a subset of genes in the heat stress response in *Arabidopsis*. *Plant Mol. Biol.* 60 759–772. 10.1007/s11103-005-5750-x 16649111

[B67] Schwarzländer M. Fricker M. D. Müller C. Marty L. Brach T. Novak J. (2008). Confocal imaging of glutathione redox potential in living plant cells. *J. Microsc.* 231 299–316. 10.1111/j.1365-2818.2008.02030.x 18778428

[B68] Silva-Correia J. Freitas S. Tavares R. M. Lino-Neto T. Azevedo H. (2014). Phenotypic analysis of the Arabidopsis heat stress response during germination and early seedling development. *Plant Methods* 10:7. 10.1186/1746-4811-10-7 24606772 PMC3975293

[B69] Sumesh K. V. Sharma-Natu P. Ghildiyal M. C. (2008). Starch synthase activity and heat shock protein in relation to thermal tolerance of developing wheat grains. *Biol. Plant* 52 749–753. 10.1007/s10535-008-0145-x

[B70] Suzuki N. Katano K. (2018). Coordination between ROS regulatory systems and other pathways under heat stress and pathogen attack. *Front. Plant Sci.* 9:490. 10.3389/fpls.2018.00490 29713332 PMC5911482

[B71] Tiwari L. D. Khungar L. Grover A. (2020). AtHsc70-1 negatively regulates the basal heat tolerance in *Arabidopsis thaliana* through affecting the activity of HsfAs and Hsp101. *Plant J.* 103 2069–2083. 10.1111/tpj.14883 32573848

[B72] Toh S. Imamura A. Watanabe A. Okamot M. Jikumaru Y. Hanada A. (2008). High temperature-induced abscisic acid biosynthesis and its role in the inhibition of gibberellin action in *Arabidopsis* seeds. *Plant Physiol.* 146 1368–1385. 10.1104/pp.107.113738 18162586 PMC2259091

[B73] Ugalde J. M. Fuchs P. Nietzel T. Cutolo E. Vothknecht U. C. Holuigue L. (2020). Chloroplast-derived photo-oxidative stress causes changes in H_2_O_2_ and EGSH in other subcellular compartments. *bioRxiv*. 10.1101/2020.07.20.212670 PMC8154069 33793922

[B74] Van Aken O. Whelan J. (2012). Comparison of transcriptional changes to chloroplast and mitochondrial perturbations reveals common and specific responses in Arabidopsis. *Front. Plant Sci.* 3:281. 10.3389/fpls.2012.00281 23269925 PMC3529323

[B75] Vogel M. O. Moore M. König K. Pecher P. Alsharafa K. Lee J. (2014). Fast retrograde signaling in response to high light involves metabolite export, MITOGEN-ACTIVATED PROTEIN KINASE6, and AP2/ERF transcription factors in *Arabidopsis*. *Plant Cell* 26 1151–1165. 10.1105/tpc.113.121061 24668746 PMC4001375

[B76] Wahid A. Gelani S. Ashraf M. Foolad M. R. (2007). Heat tolerance in plants: an overview. *Environ. Exp. Bot.* 61 199–223. 10.1016/j.envexpbot.2007.05.011

[B77] Wang Q. Chen J. He N. Guo F. (2018). Metabolic reprogramming in chloroplasts under heat stress in plants. *Int. J. Mol. Sci.* 19:849. 10.3390/ijms19030849 29538307 PMC5877710

[B78] Warland J. S. McDonald M. R. McKeown A. M. (2006). Annual yields of five crops in the family Brassicaceae in southern Ontario in relation to weather and climate. *Can. J. Plant Sci.* 86 1209–1215.

[B79] Waszczak C. Akter S. Jacques S. Huang J. Messens J. Van Breusegem F. (2015). Oxidative post-translational modifications of cysteine residues in plant signal transduction. *J. Exp. Bot.* 66 2923–2934. 10.1093/jxb/erv084 25750423

[B80] Wise R. R. Olson A. J. Schrader S. M. Sharkey T. D. (2004). Electron transport is the functional limitation of photosynthesis in field-grown Pima cotton plants at high temperature. *Plant Cell Environ.* 27 717–724. 10.1111/j.1365-3040.2004.01171.x

[B81] Wu F. Chi Y. Jiang Z. Xu Y. Xie L. Huang F. (2020). Hydrogen peroxide sensor HPCA1 is an LRR receptor kinase in *Arabidopsis*. *Nature* 578 577–581. 10.1038/s41586-020-2032-3 32076270

[B82] Zhang L. R. Li Y. L. Xing D. Gao C. J. (2009). Characterization of mitochondrial dynamics and subcellular localization of ROS reveal that *HsfA2* alleviates oxidative damage caused by heat stress in *Arabidopsis*. *J. Exp. Bot.* 60 2073–2091. 10.1093/jxb/erp078 19342427

[B83] Zhang R. Sharkey T. D. (2009). Photosynthetic electron transport and proton flux under moderate heat stress. *Photosynth. Res.* 100 29–43. 10.1007/s11120-009-9420-8 19343531

[B84] Zhou J. Xia X. J. Zhou Y. H. Shi K. Chen Z. Yu J. Q. (2014). RBOH1-dependent H2O2 production and subsequent activation of MPK1/2 play an important role in acclimation-induced cross tolerance in tomato. *J. Exp. Bot.* 65 595–607. 10.1093/jxb/ert404 24323505 PMC3904713

[B85] Zhu J.-K. (2009). Active DNA demethylation mediated by DNA glycosylases. *Annu. Rev. Genet.* 43 143–166. 10.1146/annurev-genet-102108-134205 19659441 PMC3137514

