# Supplementary figures and images for: Heat-Induced Oxidation of the Nuclei and Cytosol
Source: Front Plant Sci. 2021 Jan 12;11:617779. doi: 10.3389/fpls.2020.617779 (PMC7835529; doi:10.3389/fpls.2020.617779)

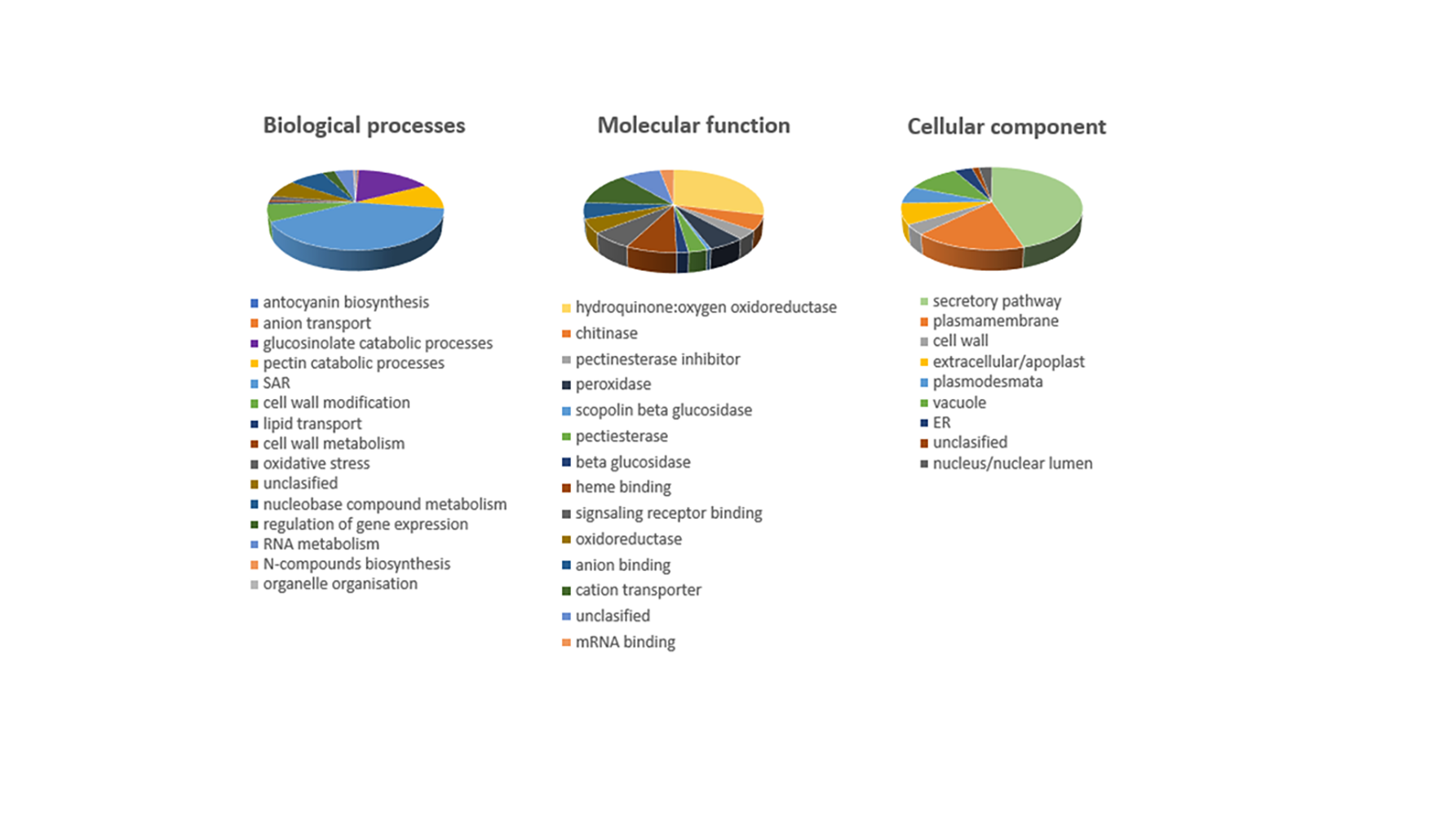

Supplement: Supplementary Figure 1 — Pie diagrams of transcripts showing heat-induced increases in abundance according to GO enrichment. All RNAs were analyzed for their respective Gene Ontology (GO) terms and fold enrichment through the GO consortium and PANTHER classification system, using Arabidopsis thaliana as a reference genome. GO enrichment that is over-represented in up-regulated set of transcripts was analyzed using to the PANTHER Classification System which contains up-to-date GO annotation data for Arabidopsis and other plant species (https://arabidopsis.org/tools/go_term_enrichment.jsp). Pie diagrams represent GO-terms for biological processes, molecular functions, and cellular components. [file Image_1.TIF]

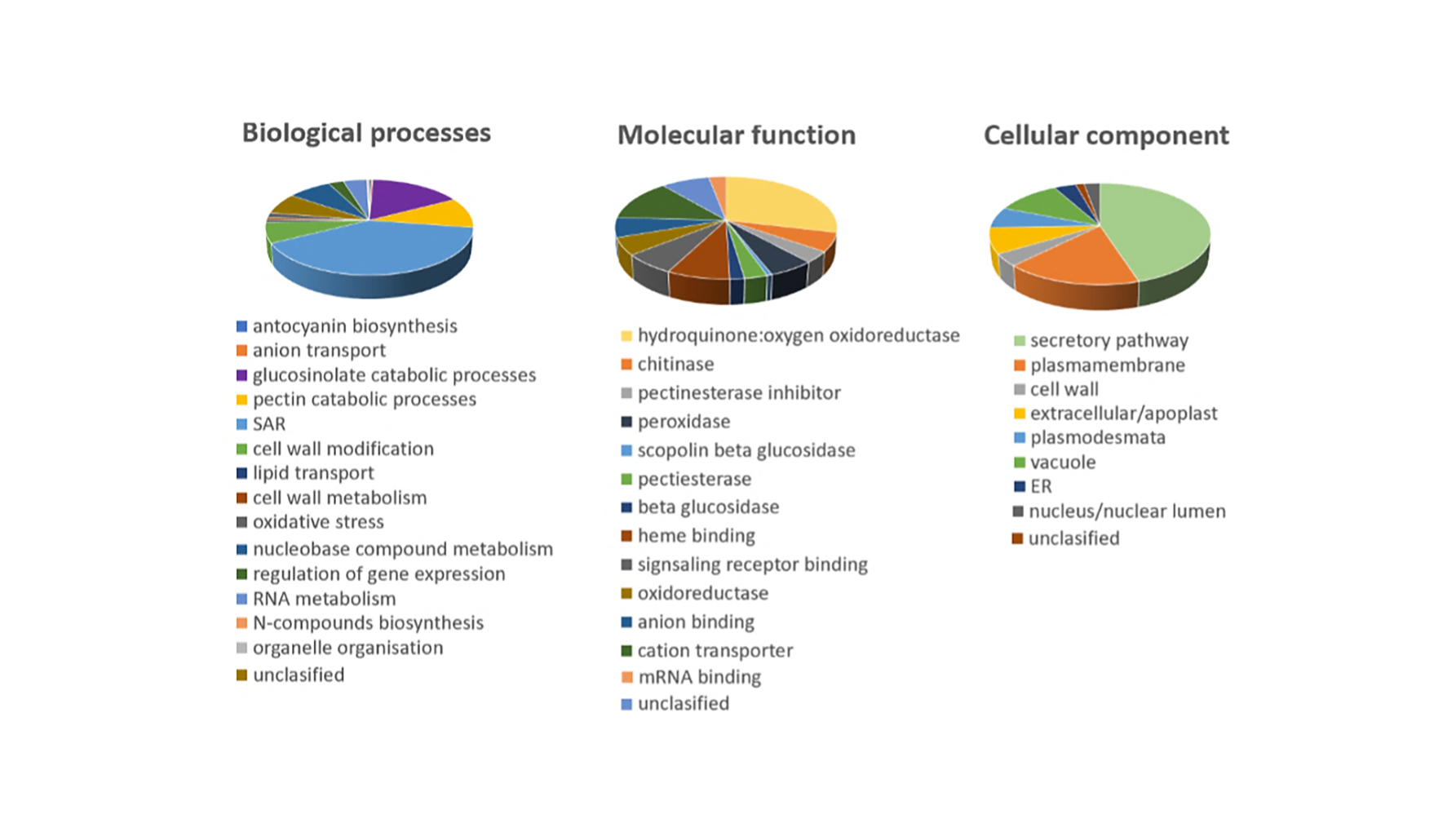

Supplement: Supplementary Figure 2 — Pie diagrams of transcripts showing heat-induced decreases in abundance according to GO enrichment for biological processes, molecular functions, and cellular components. All RNAs were analyzed for their respective gene ontology (GO) terms and fold enrichment through the GO consortium and PANTHER classification system, using Arabidopsis thaliana as a reference genome. GO enrichment that is over-represented in up-regulated set of transcripts was analyzed using to the PANTHER Classification System which contains up-to-date GO annotation data for Arabidopsis and other plant species (https://arabidopsis.org/tools/go_term_enrichment.jsp). Pie diagrams represent GO-terms for biological processes, molecular functions, and cellular components. [file Image_2.TIF]

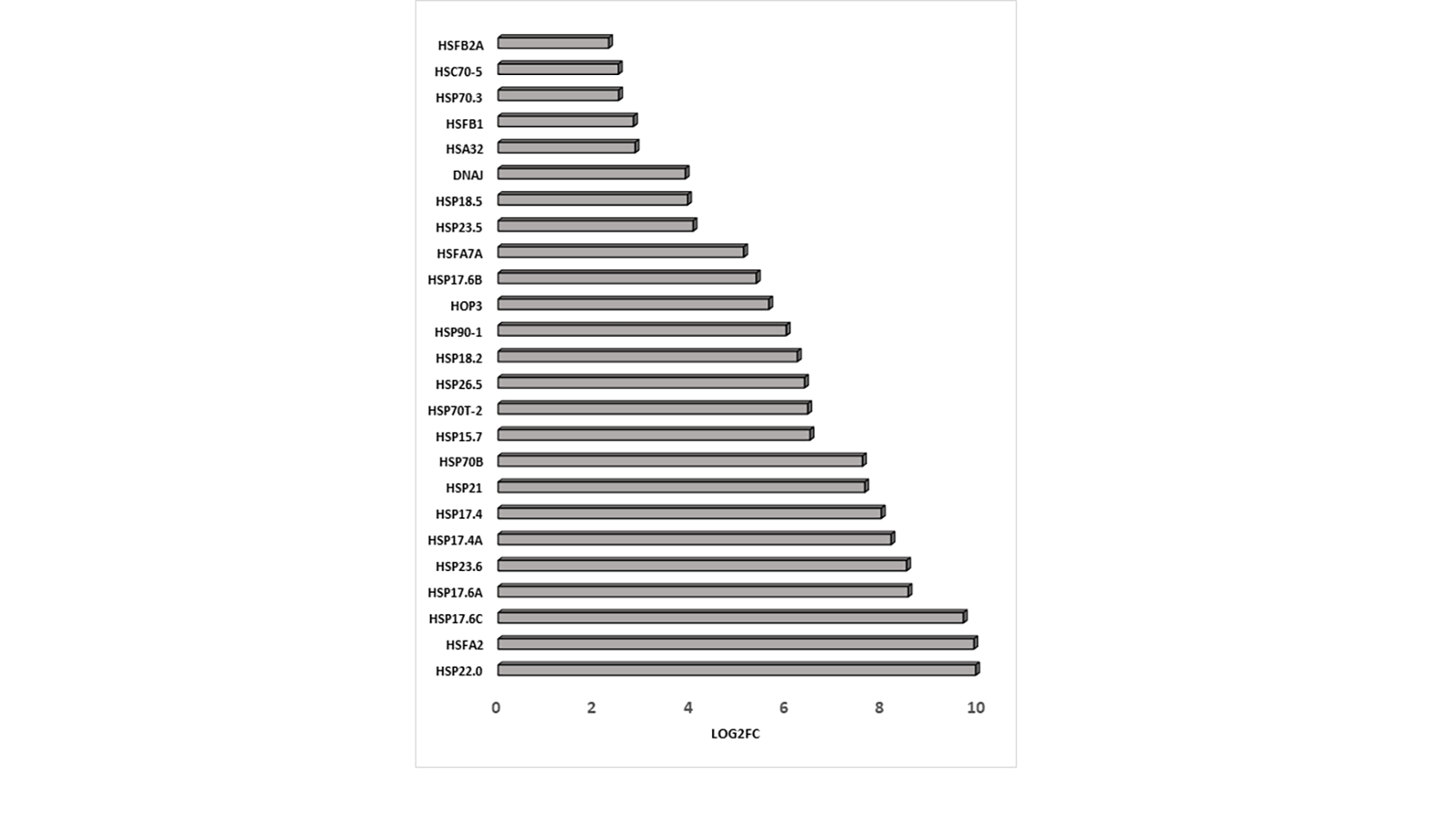

Supplement: Supplementary Figure 3 — Transcripts encoding heat shock proteins and heat shock factors that are differentially changed in abundance in response to heat stress. [file Image_3.TIF]
