# Supplementary material for: Heat-Induced Oxidation of the Nuclei and Cytosol
Source: Front Plant Sci. 2021 Jan 12;11:617779. doi: 10.3389/fpls.2020.617779 (PMC7835529; doi:10.3389/fpls.2020.617779)
Supplement: Supplementary file 5 [file Data_Sheet_1.PDF]

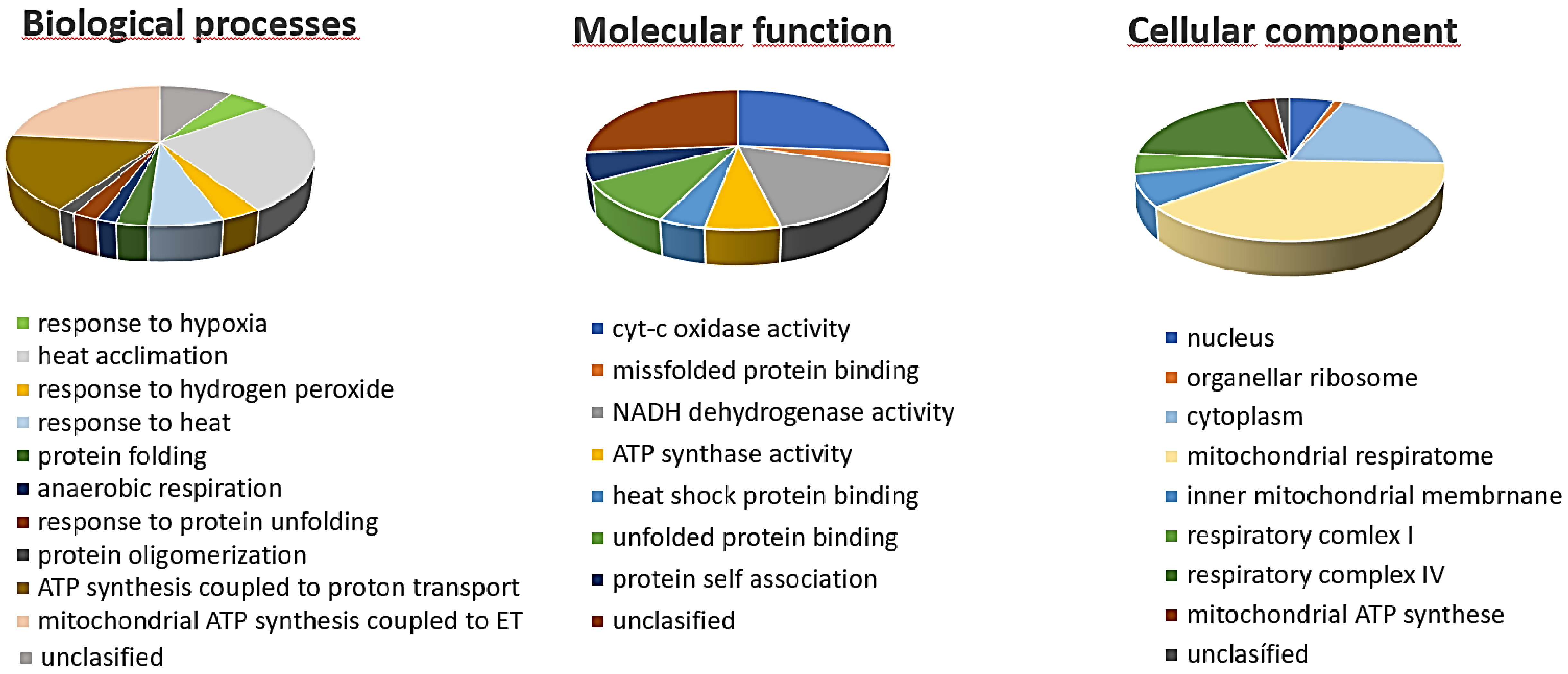
Biological processes
response to hypoxia
heat acclimation
response to hydrogen peroxide
response to heat
protein folding
anaerobic respiration
response to protein unfolding
protein oligomerization
ATP synthesis coupled to proton transport
mitochondrial ATP synthesis coupled to ET
unclasified
Molecular function
cyt-c oxidase activity
missfolded protein binding
NADH dehydrogenase activity
ATP synthase activity
heat shock protein binding
unfolded protein binding
protein self association
unclasified
Cellular component
nucleus
organellar ribosome
cytoplasm
mitochondrial respiratome
inner mitochondrial membrnane
respiratory comlex I
respiratory complex IV
mitochondrial ATP synthese
unclasífied
