# Supplementary material for: Heat-Induced Oxidation of the Nuclei and Cytosol
Source: Front Plant Sci. 2021 Jan 12;11:617779. doi: 10.3389/fpls.2020.617779 (PMC7835529; doi:10.3389/fpls.2020.617779)
Supplement: Supplementary file 6 [file Data_Sheet_2.PDF]

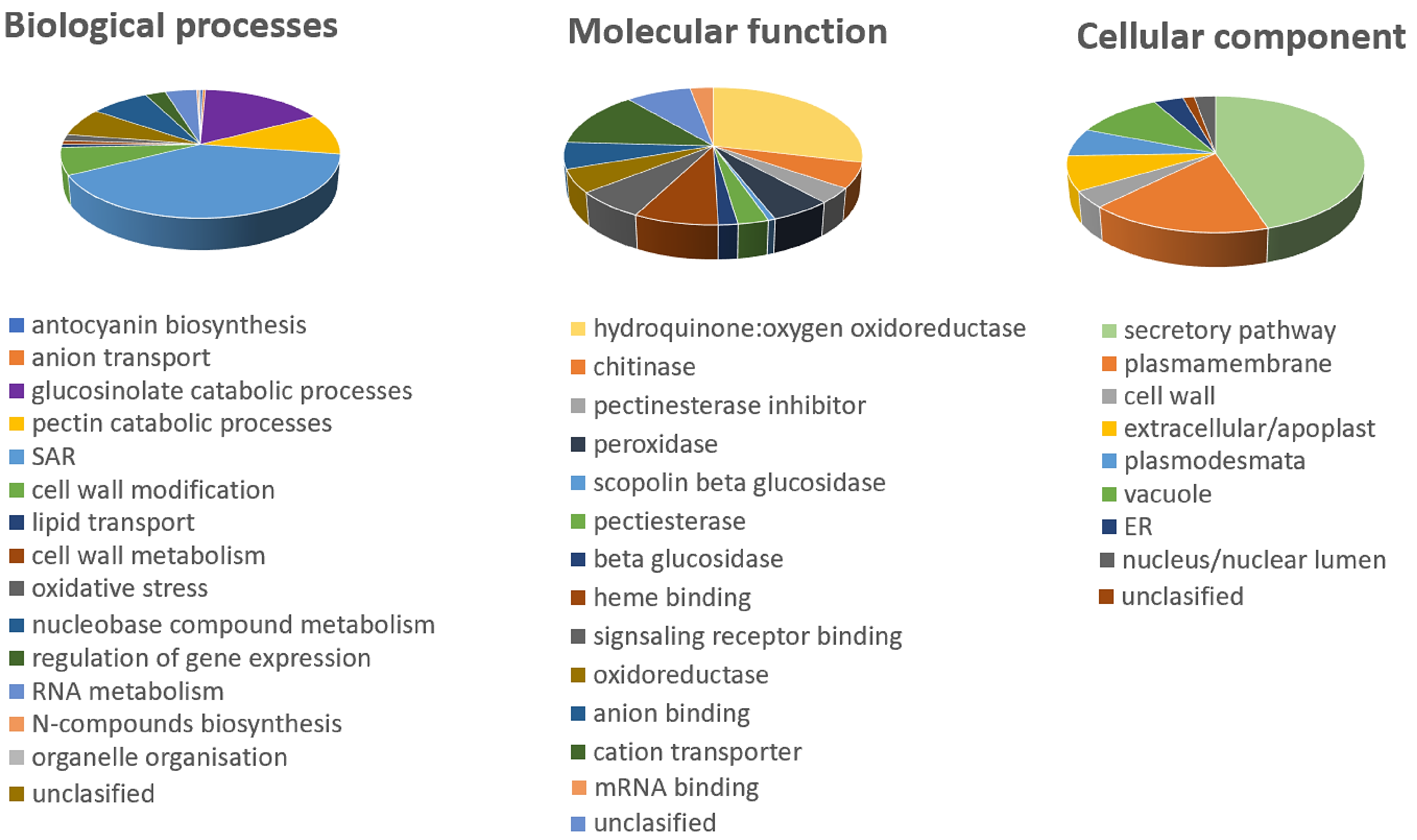
Biological processes
antocyanin biosynthesis
anion transport
glucosinolate catabolic processes
pectin catabolic processes
SAR
cell wall modification
lipid transport
cell wall metabolism
oxidative stress
nucleobase compound metabolism
regulation of gene expression
RNA metabolism
N-compounds biosynthesis
organelle organisation
unclasified
Molecular function
hydroquinone:oxygen oxidoreductase
chitinase
pectinesterase inhibitor
peroxidase
scopolin beta glucosidase
pectiesterase
beta glucosidase
heme binding
signsaling receptor binding
oxidoreductase
anion binding
cation transporter
mRNA binding
unclasified
Cellular component
secretory pathway
plasmamembrane
cell wall
extracellular/apoplast
plasmodesmata
vacuole
ER
nucleus/nuclear lumen
unclasified
